# Credit to Whom Credit Is Due

**Published:** 1888-01

**Authors:** 


### CREDIT TO WHOM CREDIT IS DUE.

We desire courteously to call the attention of the editor of *The British Journal of Dental Science* to the fact that the series of articles which he is publishing in that valuable periodical, under the title of "Contributions to the History of the Development of the Teeth," are the property of THE INDEPENDENT PRACTITIONER, and the common code of ethics which should govern all professional men demands that if he uses them he should, at the least, acknowledge the ownership. We are well aware that at the outset he gave credit, but we submit that this hardly answers the requirements of such a long series. We do not desire to be captious, but the *B. J.*

*D. S.* does not, like some American journals, live almost exclusively by general pillage, and we look to it for an example in journalistic ethics. Would it not be the proper thing to append the name of this Journal to each of the articles?

